# Relationship of spot urine oxalate to creatinine ratio and 24 hours urinary oxalate excretion in patients with urolithiasis^☆^

**DOI:** 10.1016/j.amsu.2020.11.002

**Published:** 2020-11-07

**Authors:** Syed Bilal Hashmi, Lena Jafri, Hafsa Majid, Jamsheer Talati, Wajahat Aziz, Aysha Habib Khan

**Affiliations:** a Section of Chemical Pathology, Department of Pathology & Laboratory Medicine, Aga Khan University, Pakistan; b Section of Urology, Department of Surgery, Aga Khan University, Pakistan

**Keywords:** 24-H urinary oxalate, Spot oxalate to creatinine ratio, Hyperoxaluria, Urolithiasis, HCL, Hydrochloric acid, CAP, College of American Pathologists, SPSS, Statistical Package for Social Sciences, PCNL, Percutaneous nephrolithotomy, FTIR, Fourier Transform Infrared Spectroscopy

## Abstract

**Background:**

The evaluation of 24 h urinary oxalate excretion is the gold standard for diagnosing hyperoxaluria in patients with recurrent urolithiasis. However, 24 h urine sample collection is cumbersome. Therefore we aim to see if oxalate to creatinine ratio in random urine sample can be used as an alternative.

**Materials and methods:**

A cross-sectional study was conducted at Section of Chemical Pathology, Department of Pathology and Laboratory Medicine Aga Khan University Karachi from 1st February to December 31, 2019. A total of 62 adult patients, 18–60 years of age with history of kidney stones presenting to the clinical laboratory for 24 h urine oxalate estimation were invited to participate in the study after informed consent. Clinical details were recorded on a structured questionnaire and patients were guided to submit 24 h urine and a random spot urine sample. Urinary oxalate was measured on Micro lab 300 using a kit based on oxalate oxidase principle by Trinity Biotech plc, Wicklow, Ireland following standard operating procedures. Urinary creatinine was measured on ADVIA 1800 by Siemens, US using kinetic Jaffe reaction according to the manufacturer's instructions. The data was analyzed on SPSS.

**Results:**

In a period of ten months, a total of 62 subjects were recruited; mean age was 32.4 ± 2.6 years. Males were 49 (79.0%) and females were 13 (20.9%). Correlation was found to be (r = 0.289) by Spearman correlation (p value < 0.005). Taking 24 h urinary oxalate as gold standard the sensitivity, specificity, positive predictive value and negative predictive value of spot oxalate to creatinine ratio was 83.3%, 17.8%, 9.8% and 90.9% respectively.

**Conclusion:**

The random spot urine test cannot replace the 24 h urinary oxalate estimation in patients with urolithiasis.

## Highlights

• Oxalate to creatinine ratio is proposed as an alternative to 24 h urine.
• Sensitivity & specificity of spot ox:cr ratio was 83.3% and 17.8% respectively.
• PPV and NPV of spot ox:cr ratio was 9.8% and 90.9% respectively.
• Studies taking diet and molecular testing into consideration are needed.

Oxalate is a metabolic end product of glyoxalate metabolism excreted by the kidneys. It has a high affinity for calcium and form calcium oxalate crystals, which has low solubility and are typically deposited within the renal interstitium and tubular cells [1]. Hyperoxaluria may present with recurrent kidney stones to end stage renal disease and systemic oxalosis due to reduced urinary excretion when glomerular filtration rate (GFR) falls below 30–40 mL/min per 1.73 m^2^ [2].

The evaluation of 24 h urinary oxalate excretion is considered gold standard for diagnosing hyperoxaluria in patients with recurrent urolithiasis [3]. However, 24 h urine sample collection is cumbersome and often inaccurate due to factors such as the patient missing a urine pass in the collection jar, the quantity of acid added preservative to the container or the possibility of other liquid being added to the container instead of urine to fulfill the volume requirement [4]. In addition, a study by Parks et al. showed that principle stone risk factors and dietary and environmental factors vary enough in 24 h urine samples making a 24 h urinary sample inadequate for correct diagnosis [5]. It is proposed that a single specimen is not an ideal predictor of hyperoxaluria and the final mean of two 24 h urine samples should be taken for diagnosis. Considering these limitations 24 h urinary oxalate is usually not practical to screen for hyperoxaluria. A second morning random urine sample, for oxalate to creatinine ratio has been proposed as an alternative [6,7].

Oxalate: creatinine ratio is considered a better index of oxalate excretion as it avoids error from an inaccurately timed collection and ease of calculation from a spot urine sample. This is an advantage especially for children and severely ill patients. A correlation ‘r’ of 0.352 between 24 h urinary oxalate and spot urinary oxalate to creatinine ratio was shown in the study by Hong et al. (p value 0.028). And found that the two paired measurements did not show good agreement, based on the estimated “limit of agreement” by the Bland and Altman test of agreement [4]. The reason for the disagreement was believed to be due to the time-to-time variations in the urinary oxalate concentrations that vary according to the body hydration status, diurnal circadian rhythm and period after food intake. Seema L et al. from India reported significantly high oxalate: creatinine ratio in stone formers compared to control group (p < 0.05) on spot urine samples and spot urinary samples are recommended spot urinary samples as screening procedure for hyperoxaluria [8].

The relationship of spot and 24 h urine oxalate has not been studied in Pakistani population, where the frequency of undifferentiated hyperoxaluria is seen in up to 43% of paediatric stone formers [9]. We therefore studied the relationship between 24 h urinary oxalate excretion and spot urine oxalate to creatinine ratio to determine its clinical utility against the use of 24 h urinary oxalate estimation which is the standard practice.

## 1 Material & methods

A cross-sectional study was conducted at Section of Chemical Pathology, Department of Pathology and Laboratory Medicine, Aga Khan University, Karachi from 1st February to December 31, 2019. Adult patients with history of kidney stones presenting at the Clinical Laboratory for 24 h urine oxalate estimation were included in the study. Debilitated or bed ridden patients and patients with renal failure or end stage renal disease, in whom urinary oxalate is no longer an indicator of disease status were excluded from the study. Study was approved by ethical review committee of Aga Khan University Hospital. Patients after informed consent, were guided to submit a spot urine sample. Clinical details were recorded on a structured questionnaire by the principal investigator.

24 h urine samples were collected in a jar having thymol, which is routinely added to limit bacterial growth and hence guard against citrate degradation. Spot urine samples were collected in red top container without any added preservatives. The specimen were transported to the laboratory within the 12 h of collection of 24 h urinary specimen.

Volume of 24 h urine was measured in liters. After mixing of the urinary sample to overcome effects of sedimentation, a 6 mL aliquot was made and HCl was added to avoid the precipitation of oxalate crystals in every sample. Specimen was stored at −20C till further analysis.

Urinary oxalate was measured on Micro lab 300 using a kit based on oxalate oxidase principle by Trinity Biotech plc. Wick low, Ireland following standard operating procedures. Urinary creatinine was measured on ADVIA 1800 by Siemens, US using kinetic Jaffe reaction. Both normal and abnormal quality control materials were run with every batch of oxalate and creatinine analysis in urine to validate the results. In addition, external proficiency testing of urinary oxalate was conducted by College of American Pathologists (CAP).

The cut-offs for 24 h oxalate excretion was 502 ųmol/24 h for males and 353 ųmol/24 h for females. The cut-offs for spot oxalate: creatinine ratio was 33 ųmol/mmol for males and 45 ųmol/mmol for females [10].

### 1.1 Statistical analysis

Data was entered and analyzed in to SPSS 22 version. To check the normality of the data Shapiro Wilk test was applied. Descriptive statistics were given as mean, median and interquartile range (IQR). Frequency and percentages were calculated for gender distribution, recurrent stone, and family history of urolithiasis respectively. Spearman's correlation was applied because data was non-parametric. Concordance between 24 h and spot oxalate excretion was run and Cohen's kappa values were calculated. *P*-value ≤0.05 was taken as statistically significant.

## 2 Results

In a period of ten months, a total of 62 subjects with urolithiasis were recruited. Mean age was 32.4 ± 2.6 years. Males were 49 (79.0%) and females were 13 (20.9%).

Majority of the subjects had ureteric stones (62.5%), followed by renal and bladder stones in 28.2% & 6.3% respectively. Twenty subjects had undergone surgery for stone removal (9 had extracorporeal shock wave lithotripsy, 11 had PCNL). However, the stone analysis was available in 7 cases (35%) only, which in all cases consist of calcium oxalate crystals as the primary constituent. The stone analysis was done by Fourier-transform infrared spectroscopy (FTIR).

The most common presenting complaint was flank pain (79.7%) followed by nausea and vomiting (53.1%) and dysuria (23.4%). The risk factors pertaining to urolithiasis in the study subjects were decreased water consumption (67.2%), previous history of stones (21.9%), family history of stones (20.3%) and smoking (10.9%).

Table 1 shows the overall and gender wise distribution of median (IQR) of 24 h urinary oxalate and spot oxalate to creatinine ratio with distribution of subjects with high oxalate excretion. Six subjects (5 males and 1 female) had high 24 h urine oxalate excretion, of which 5 had high spot oxalate to creatinine ratio. Spot oxalate: creatinine ratio was high among 51 patients (42 males and 9 females). Table 2 shows the comparison of 24 h urinary oxalate and spot oxalate to creatinine ratio in 7 subjects with calcium oxalate crystals on stone analysis. Only 2 subjects had hyperoxaluria in contrast to all subjects with high spot oxalate to creatinine ratio. Taking 24 h urinary oxalate as gold standard the sensitivity, specificity, positive predictive value and negative predictive value of spot oxalate to creatinine ratio was 83.3%, 17.8%, 9.8% and 90.9% respectively in diagnosing hyperoxaluria.

**Table 1:** Comparison of overall and elevated median (IQR) of urinary oxalate excretion in 24-h & spot urine samples in both genders (n = 62).

	Median (IQR)	No of Patients with High Oxalate Value in Each Group			
		Male (n = 49)		Female (n = 13)	
		N (%)	Median (IQR)	N (%)	Median (IQR)
24 h urine oxalate in umol/24 h	217 (138.2)	5 (10.2%)	592.8 (189.2)	1 (7.6%)	370.5*
Spot urine oxalate to creatinine ratio in ųmol/mmol	63.3 (45.6)	42 (85.7%)	77.5 (48.5)	9 (69.2%)	69.3 (39.9)

For 24 h urine oxalate excretion cutoff used of 502 μmol/24 h was used for labelling Hyperoxaluria in males while in females a cutoff 353 μmol/24 h was applied. For spot urine oxalate to creatinine ratio a cutoff of 33 ųmol/mmol was used for identifying Hyperoxaluria in males while in females a cutoff 45 ųmol/mmol was applied [9].

*IQR not calculated.

**Table 2:** Comparison of 24 h urinary oxalate and spot urine oxalate to creatinine ratio in patients tested by stone analysis (n = 7).

S·NO*	Age (Years)	Gender	24 h urine oxalate (umol/24 h)	Spot urine Ox/Cr (ųmol/mmol)	Family History
1	40	Male	539.2	94.7	Present
2	29	Male	513	109	Absent
3	58	Male	228	64.5	Absent
4	26	Male	305.5	139.1	Absent
5	23	Male	216.6	83.3	Absent
6	19	Male	148.2	89	Present
7	22	Male	114	83.6	Absent

*All subjects had calcium oxalate crystals on stone analysis.

**Fig. 1:**
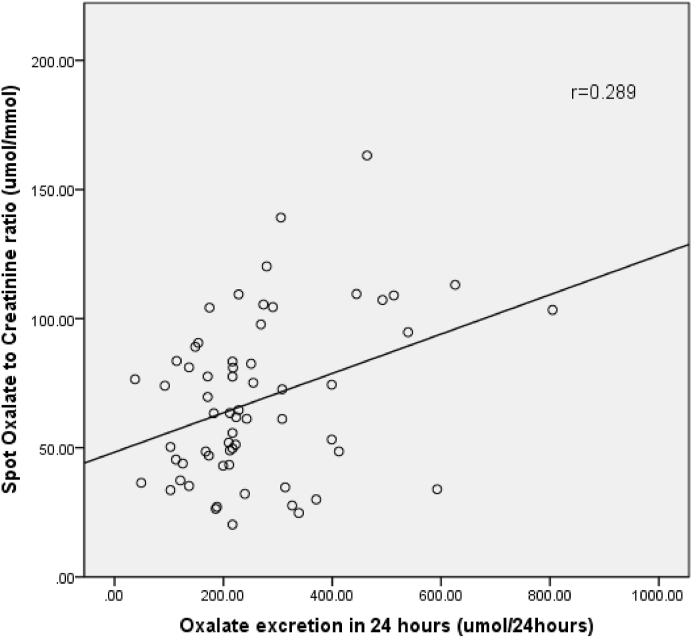
Relation of Spot oxalate to creatinine ratio to 24 h oxalate excretionCorrelation between the two tests was assessed by Spearman's correlation coefficient (r = 0.289).

Poor concordance was seen between 24 h oxalate excretion and spot oxalate to creatinine ratio in the subjects studied with a kappa value 0.037. Poor correlation (r = 0.289) was found between 24 h urine oxalate and oxalate to creatinine ratio by applying Spearmans correlation. However, it was statistically significant (p value < 0.005), as shown in Fig. 1.

## 3 Discussion

To the best of our knowledge, this is the first study about relationship between 24 h urine oxalate and spot oxalate to creatinine ratio in Pakistani population. Several studies have assessed the prospect of using spot urine or timed, urine samples for metabolic evaluation in patients with urolithiasis. Studies have reported good correlations between the 24 h urine calcium and phosphate excretions and their corresponding spot urine calcium/creatinine and phosphate/creatinine ratios but there is not much literature on oxalate comparison in 24 h and spot sample in stone formers [8,10]. Some investigators have argued on the association between the 24 h urinary excretion and their corresponding spot urine creatinine ratio as a good indicator for using the spot urine sample in replacement to the 24 h urine sample. Yet Hoi Hong investigated the correlations and agreements between the solute/creatinine ratios from the 24-h and early morning spot urine samples for metabolic evaluation in stone-formers. The investigators reported poor correlation (r = 0.352) of 24 h oxalate excretion and spot urinary oxalate: creatinine ratio. They concluded that morning spot urine is not appropriate and cannot replace the 24-h urine collection in the evaluation of urinary metabolic abnormalities in stone-formers [4]. However, the correlation coefficient may not be the appropriate statistical test in assessing whether measurements from the two methods agree with each other as in our study [11]. A high correlation does not represent a good agreement between the two related measurements because the measurement with a larger range of true quantity in the sample will have a higher correlation, in which solute/creatinine ratio of a single urinary parameter can vary widely in range, thus ensuring a significant correlation. The reason for the disagreement is believed to be mainly due to the time-to-time variations in the urinary solute concentrations (urinary volume) that vary according to the body hydration status, diurnal circadian rhythm and dietary patterns affecting oxalate excretion [12].

Primary hyperoxaluria or oxalosis is associated with recurrent renal stone formation that usually begins in childhood [11]. Therefore in patients with recurrent renal calculi or unexplained renal failure primary hyperoxaluria must be considered as an important, though rare diagnosis. Prompt diagnosis and appropriate management are crucial. For screening and accurate identification of primary hereditary hyperoxaluria or secondary hyperoxaluria it is essential to quantitate urinary oxalate [13]. As daily urine collection for determining oxalate excretion is cumbersome we studied the relation of daily oxalate excretion with oxalate: creatinine ratio in randomly collected urine sample. In the stone formers studied, only six patients out of the total sixty two patients showed elevated urinary oxalate excretion however the spot oxalate: creatinine ratio was high in 51 patients. Poor concordance was seen between 24 h oxalate excretion and spot oxalate to creatinine ratio in the subjects studied with a kappa value 0.037. Considering twenty four urinary oxalate excretion as the gold standard the study shows hyperoxaluria in 9.6% (n = 6) of the patients studied.

### 3.1 Limitations

The limitations in our study included single centre study and small sample size. Also the dietary habits, environmental conditions, body composition were not assessed.

## 4 Conclusion

In conclusion, the random spot urine test cannot replace the 24 h urinary oxalate estimation in patients with urolithiasis. Further studies should be conducted taking diet, water intake, drugs and comorbidity into consideration and understand the influence of diet on 24 h urine oxalate and spot urine oxalate to creatinine ratio.

## Provenance and peer review

Not commissioned, externally peer-reviewed.

## Funding

Resident Research Grant Committee of Rs 40,000 PKR.

## Ethical approval

ERC Number: 5084-Pat-ERC-17.

## Consent

A copy of the written consent will be available for review by the Editor-in-Chief of this journal on request.

## Author contribution

SBH, AHK & WA: Conception, design of study and manuscript writing.

JT & AHK: Drafting the work and revising it critically for important intellectual content. Also final approval of version to be published.

LJ, HM & SBH: Data collection and literature review.

## Registration of research studies

1. Name of the registry: Clinicaltrials. gov.

2. Unique Identifying number or registration ID: NCT04571359.

3. Hyperlink to your specific registration (must be publicly accessible and will be checked): https://register.clinicaltrials.gov/prs/app/action/SelectProtocol?sid=S000AA0V&selectaction=Edit&uid=U0005AWR&ts=4&cx=-ahw23v.

## Guarantor

Dr. Aysha Habib Khan.

## Declaration of competing interest

None.
